# Refractive index tomograms and dynamic membrane fluctuations of red blood cells from patients with diabetes mellitus

**DOI:** 10.1038/s41598-017-01036-4

**Published:** 2017-04-21

**Authors:** SangYun Lee, HyunJoo Park, Kyoohyun Kim, YongHak Sohn, Seongsoo Jang, YongKeun Park

**Affiliations:** 1 Department of Physics, Korea Advanced Institute of Science and Technology, Daejeon, 34141 Republic of Korea; 2 Department of Laboratory Medicine, Eulji University Hospital, Daejeon, 35233 Republic of Korea; 3 Department of Laboratory Medicine, Asan Medical Center, University of Ulsan, College of Medicine, Seoul, 05505 Republic of Korea; 4 Tomocube Inc., Daejeon, 34051 Republic of Korea

## Abstract

In this paper, we present the optical characterisations of diabetic red blood cells (RBCs) in a non-invasive manner employing three-dimensional (3-D) quantitative phase imaging. By measuring 3-D refractive index tomograms and 2-D time-series phase images, the morphological (volume, surface area and sphericity), biochemical (haemoglobin concentration and content) and mechanical (membrane fluctuation) parameters were quantitatively retrieved at the individual cell level. With simultaneous measurements of individual cell properties, systematic correlative analyses on retrieved RBC parameters were also performed. Our measurements show there exist no statistically significant alterations in morphological and biochemical parameters of diabetic RBCs, compared to those of healthy (non-diabetic) RBCs. In contrast, membrane deformability of diabetic RBCs is significantly lower than that of healthy, non-diabetic RBCs. Interestingly, non-diabetic RBCs exhibit strong correlations between the elevated glycated haemoglobin in RBC cytoplasm and decreased cell deformability, whereas diabetic RBCs do not show correlations. Our observations strongly support the idea that slow and irreversible glycation of haemoglobin and membrane proteins of RBCs by hyperglycaemia significantly compromises RBC deformability in diabetic patients.

## Introduction

Diabetes mellitus is a life-threatening disease affecting many people. Consequences of all diabetes types include chronically elevated blood glucose levels, i.e. hyperglycaemia, caused by a lack of insulin or loss of insulin-mediated metabolic functions, which eventually results in a wide range of complications ranging from microvascular diseases including retinopathy, neuropathy and nephropathy, and cardiovascular diseases, to severe depression and dementia^1^.

Characterisation of the deformability of red blood cells (RBCs) is crucial to understanding the pathophysiology of diabetes because it is strongly related to the disease’s complications. The deformability of diabetic RBCs has been extensively investigated using various experimental approaches including optical microscopy^2^, Coulter counter^3^, and laser diffraction ektacytometry^4^. In particular, the main cytoplasmic alteration in diabetic RBCs caused by hyperglycaemia, the glycation of haemoglobin (Hb), has gained increasing attention because the relative amount of glycated Hb, mainly consisting of HbA1c to total Hb, reflects the mean plasma glucose concentration of the previous three months^5^. Also, the glycosylation of other intracellular and membrane proteins of diabetic RBCs has been reported^6^. Furthermore, damages to RBC membranes resulting from chronic hyperglycaemia have been accessed by measuring end products of lipid-peroxidation^7, 8^. It has been known that diabetic RBCs exhibit significantly affected deformability compared to non-diabetic RBCs using micropipette aspiration^9^, ektacytometry^10^, fabrication of micro-channels^11^ and membrane filters mimicking capillary vessels^12^. Instant effects of *in vitro* glucose treatments on RBC deformability have been addressed^13^.

These investigative approaches have significantly enhanced our understanding of the rheology of diabetic RBCs. However, none of these approaches can simultaneously probe morphological, biochemical and mechanical parameters, particularly at the individual cell level. Moreover, such previous approaches depend on large external loads or deformation of RBCs via physical contact, and thus are not well suited to precise measurement of soft and elastic properties of RBCs within linear deformation regimes. Accordingly, previous approaches do not allow systematic correlative analyses to be performed on simultaneously measured various cellular parameters. As a consequence, a systematic study on the integrated effects of diabetic complications or hyperglycaemia on individual human RBCs has not been fully investigated previously.

To effectively address the existing problems, we present a systematic study measuring individual parameters in RBCs from patients with diabetes using the quantitative phase imaging (QPI) technique^14, 15^. QPI is an interferometric imaging technique which can precisely and quantitatively measure live cells and tissues, utilising the refractive index (RI) as an intrinsic optical contrast. Recently, QPI techniques have been utilised for the study of various biological and medical areas, including malarial infection^16–18^, haematology^19, 20^, neuroscience^21^ and sickle cell disease^22, 23^. In addition, QPI has also been employed as a tool for three-dimensional (3-D) cell morphometry^24^ or to reveal the capability of single RBC as an adaptive optofluidic microlens^25^. Using this QPI technique, we performed label-free and quantitative measurements of live RBCs, and assessed 3-D refractive index (RI) tomograms and 2-D time-series phase maps of individual cells. From the measured 3-D RI tomograms and the time-series phase images, the morphological (volume, surface area and sphericity), biochemical (Hb concentration and Hb content) and mechanical (membrane fluctuation) parameters were retrieved. Also, correlative analyses among six measured RBC parameters were systematically performed and analysed with the information about independently measured HbA1c.

## Results

To reconstruct the 3-D morphologies of individually measured RBCs, we employed a QPI technique called common-path diffraction optical tomography (cDOT)^26, 27^. Employing the principle of optical diffraction tomography^28–30^, cDOT measures 3-D RI tomograms of cells and tissues, which is optically analogous to X-ray computed tomography (For details, see *Materials and Methods*). Multiple 2-D holograms of a sample were recorded with various angles of laser illumination, from which complex optical fields of light at the sample plane were retrieved using a field retrieval algorithm^31^. Then, from a set of the retrieved multiple 2-D optical fields, a 3-D RI distribution of the sample was reconstructed.

**Figure 1:**
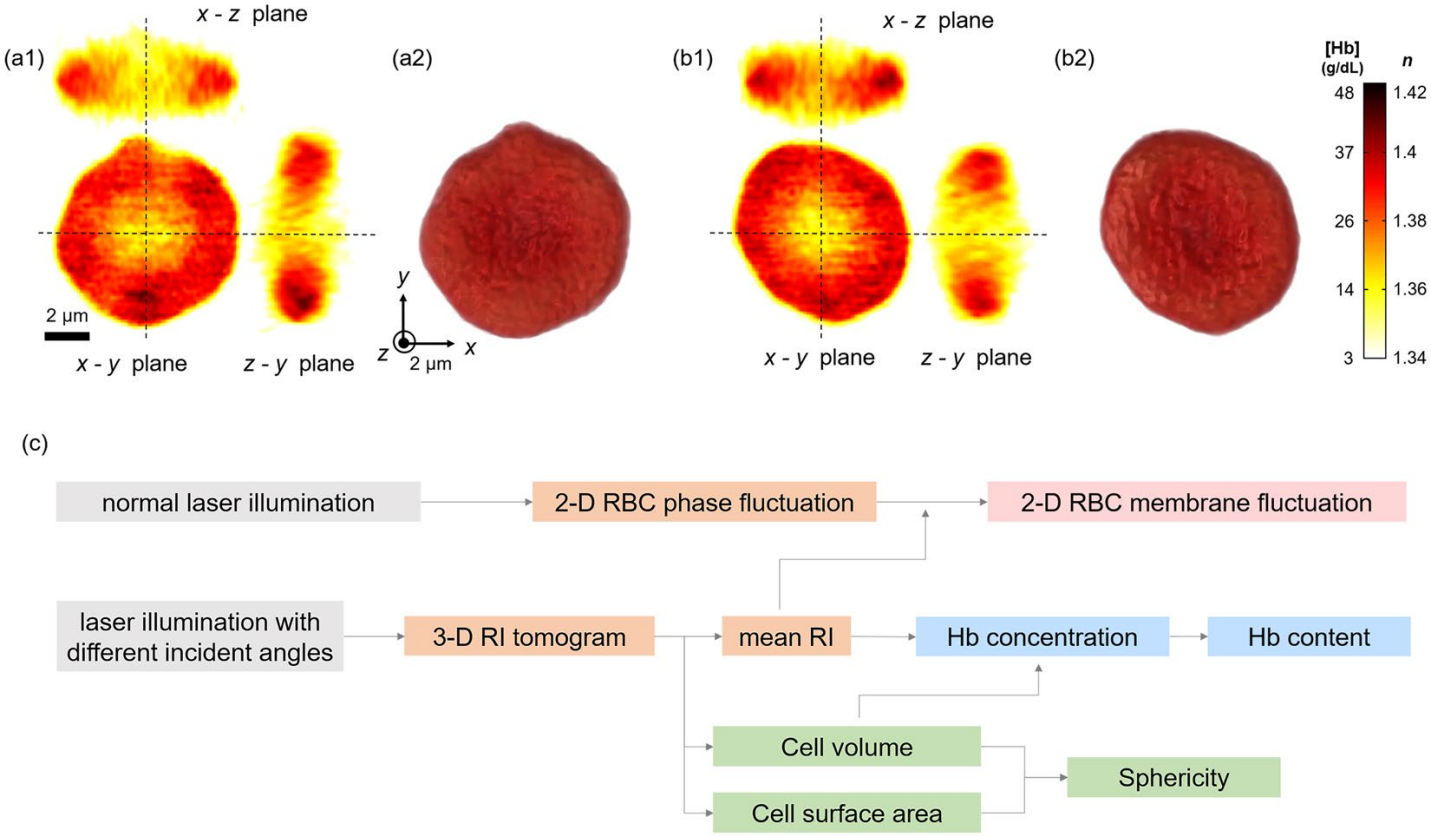
(**a**,**b**) Reconstructed 3-D RI tomograms and isosurface renderings of representative non-diabetic and diabetic RBCs. Cross-sectional images (along the *x*-*y*, the *z*-*y* and the *x*-*z* planes) of the 3-D RI tomogram of the representative non-diabetic (**a1**) and diabetic (**b1**) RBCs. RI isosurfaces (*n* > 1.35) of the corresponding non-diabetic (**a2**) and diabetic (**b2**) RBCs were presented. (**c**) Flow diagram for retrieval of six RBC parameters.

Using cDOT, we measured 3-D RI tomograms of 40 RBCs per person for a total of 12 non-diabetic and diabetic blood donors (see *Materials and Methods*). The reconstructed 3-D RI tomograms and the RI isosurfaces (〈*n*〉 > 1.35) of the representative non-diabetic and diabetic RBCs are presented in Fig. 1. Cross-sectional images of the measured RI tomograms of both the non-diabetic and diabetic RBCs show characteristic biconcave shapes. Because the RI value of the RBC cytoplasm is linearly proportional to the intracellular Hb concentration with a known proportionality coefficient, RI increment^32^, the RI value of RBC can be directly related to Hb concentration.

Figure 1(c) summarises the procedure required for retrieving quantitative RBC parameters (see *Materials and Methods*). The morphological parameters (cell volume and surface area) are directly obtained from the reconstructed 3-D RI tomogram. Cell sphericity is a dimensionless number, defined as a normalised volume-to-surface area ratio, which has been used for addressing the sphere-likeness of cells^33, 34^. Perfect spheres and flat disks have a sphericity of 1 and 0, respectively. When it comes to normal RBCs, it has been known that this parameter is acutely influenced by diverse physiological conditions including osmolality of plasma^35^, the storage period of blood packs^36^, and cell aging^37^. Cytoplasmic Hb concentration of RBCs is extracted from the measured RI value because RBC cytoplasm mainly consists of Hb solution, and there is a linear relationship between the RI of the Hb solution and its Hb concentration^32, 38^. Then, the retrieved Hb concentration and cell volume offer information about the cytoplasmic Hb content. While laser illumination is set to be perpendicular to an RBC, optical phase delays contributed from the cell membrane fluctuation can be continuously recorded using a high-speed camera. Finally, dynamic membrane fluctuations of individual cells are calculated from 2-D phase fluctuations and mean RI values from the reconstructed 3-D RI tomograms. Recently, it has been reported that these RBC membrane fluctuations are acutely affected by diverse pathophysiological conditions^17, 18, 22, 39–43^.

### Retrieved RBC parameters of all non-diabetic and diabetic blood donors

To address the morphological and biochemical alterations in human RBCs by diabetes mellitus, we retrieved three morphological (volume, surface area and sphericity) and two biochemical (cytoplasmic Hb concentration and Hb content) RBC parameters for every measured RBC from its 3-D RI tomogram.

**Figure 2:**
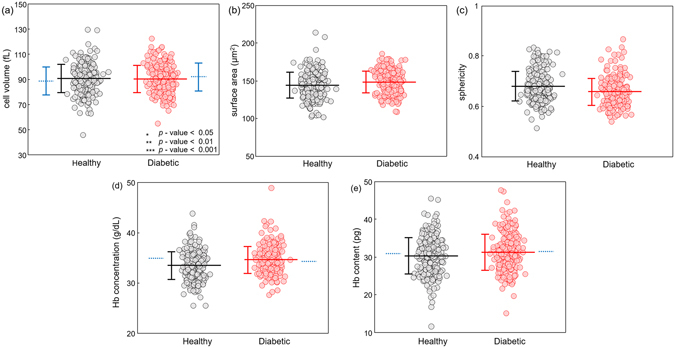
Morphological and biochemical parameters for all measured RBCs from healthy controls and diabetic patients: (**a**) volume, (**b**) surface area, (**c**) sphericity, (**d**) cytoplasmic Hb concentration and (**e**) Hb content. Each circle denotes an individual RBC measurement. The horizontal lines and the error bars represent the calculated mean value and sample standard deviation, respectively. The blue dotted lines correspond to results from complete blood count tests.

As shown in Fig. 2(a), there is no statistical difference in volume distributions of non-diabetic and diabetic RBCs. The mean volumes of non-diabetic and diabetic RBCs are 90.5 ± 11.4 [mean ± standard deviation (SD)] and 90.2 ± 10.9 fL, respectively. Diabetic RBCs also exhibit no statistically different surface area compared to that of non-diabetic RBCs. The mean surface areas of non-diabetic and diabetic RBCs are 144.1 ± 17.4 and 148.2 ± 14.4 μm^2^, respectively. The calculated mean sphericity values of non-diabetic and diabetic RBCs are 0.68 ± 0.06 and 0.66 ± 0.05, respectively.

The retrieved Hb concentration and the Hb content of non-diabetic and diabetic RBCs are presented in Fig. 2(d) and (e), respectively. The calculated mean Hb concentration of non-diabetic and diabetic RBCs are 33.4 ± 2.8 and 34.6 ± 2.7 g/dL, respectively. Despite the slightly elevated mean Hb concentration in diabetic RBCs, performed Wilcoxon rank-sum test does not guarantee the statistical difference with a *p*-value of 0.17. The cytoplasmic Hb content of diabetic RBCs is also not statistically different with that of the control RBCs. The retrieved mean Hb content in healthy and diabetic RBC cytoplasm is 30.3 ± 4.8 and 31.2 ± 4.8 pg, respectively. Our optical measurements on cytoplasmic Hb content agree well with the mean corpuscular Hb values of blood donors which were independently measured using the complete blood count machine (the blue dotted lines in Fig. 2). The retrieved RBC parameters of individual subjects with both complete blood count (CBC) result sheet and independently determined HbA1c level are presented in the Supplementary Information (see Supplementary Figure S1).

### Alterations in mechanical property of RBCs by diabetes mellitus

Cell deformability represents the ability of a cell to change and restore its shape in response to external stimuli. In particular, the remarkably high deformability of RBCs has a core role in transporting oxygen to body organs and tissues through capillaries with diameters even smaller than those of RBCs. It has been widely accepted that the main factors governing the overall RBC deformability include the cell sphericity, mechanical properties of membranes, and cytoplasmic viscosity^44, 45^, which are mainly affected by alterations in cell volume or surface area, cytoskeletal spectrin networks, and intracellular Hb concentrations, respectively.

To investigate the mechanical alterations in RBC membranes by diabetes, we measured dynamic membrane fluctuations of non-diabetic and diabetic RBCs. Recently, it has been shown that QPI techniques can be used to precisely probe dynamic fluctuations in cell membranes^46^, and these have been utilised in diverse applications in infectious disease^17, 42^, sickle cell disease^47^, and haematology^19, 48^.

**Figure 3:**
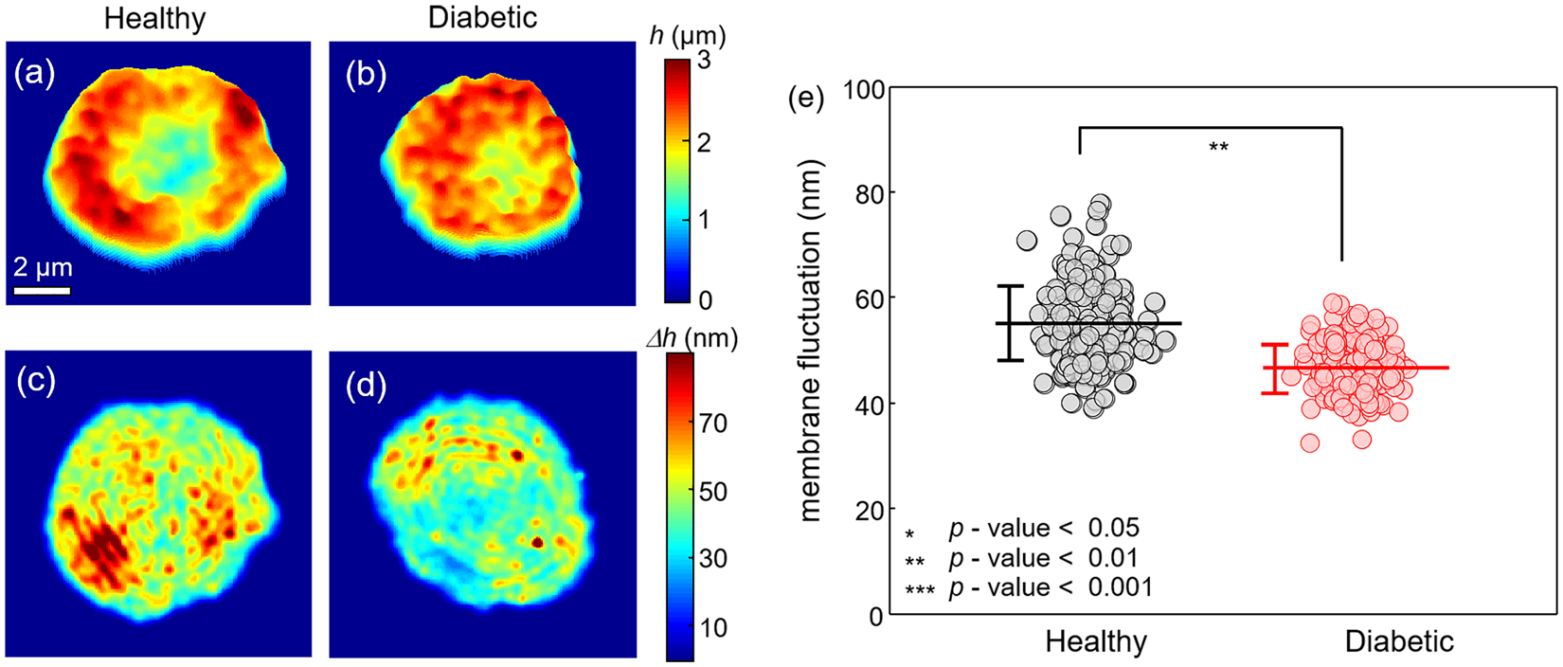
2-D membrane height maps of the representative RBCs from (**a**) healthy controls and from (**b**) diabetic patients, respectively. 2-D membrane fluctuation maps of the corresponding RBCs are represented in (**c**) and (**d**). (**e**) Graph for retrieved membrane fluctuations of every measured RBC. The horizontal lines and the vertical error bars denote the mean value and sample standard deviation of the measured RBC membrane fluctuations, respectively.

The 2-D topographic height maps of representative non-diabetic and diabetic RBCs are shown in Fig. 3(a) and (b), respectively. The corresponding dynamic membrane fluctuation maps, defined as temporal SDs of the RBC height profiles, are also presented in Fig. 3(c) and (d) (*Materials and Methods*).

To investigate cell deformability, we spatially averaged the 2-D membrane fluctuation map of individual RBCs and defined it as the mean membrane fluctuation. As shown in Fig. 3(e), it is clear that diabetic RBCs exhibit significantly decreased mean membrane fluctuations compared to healthy RBCs, implying diminished deformability of diabetic RBCs (*p*-value < 0.01). The averaged values of mean membrane fluctuations for non-diabetic and diabetic RBCs are 55.1 ± 6.9 and 46.6 ± 4.5 nm, respectively. These observed diminutions of membrane fluctuation in diabetic cells are qualitatively in accordance with previous studies on decreased RBC deformability by diabetes based on micropipette aspiration^9^, laser diffraction ektacytometry^10, 49^, and techniques using optical tweezers^50^ and carbonate membrane filters^12^.

### Membrane fluctuations of RBCs in relation to HbA1c level

HbA1c reflects the degree of hyperglycaemia. HbA1c level is defined as a percentage of HbA1c, the majority of glycated Hbs in RBC, to the total Hb content in blood. Because HbA1c is formed over 120 days, non-enzymatic glycation process of Hb^51, 52^, the HbA1c level has been perceived as an effective means to estimate the mean glucose concentration of individuals for the previous three months^5, 53^.

**Figure 4:**
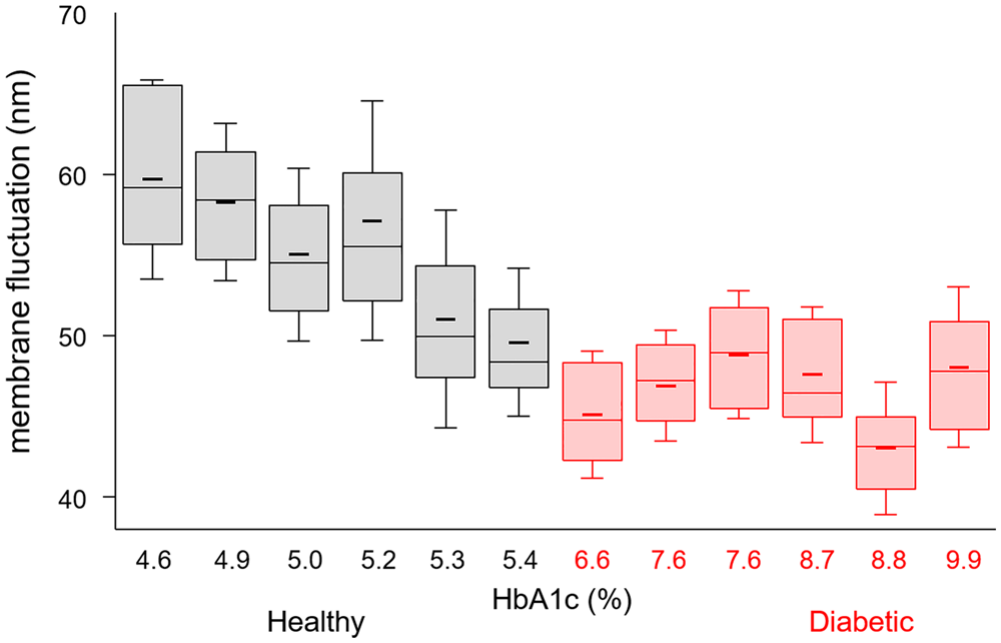
Box plots of membrane fluctuations for measured RBCs from six healthy controls and six diabetic patients in increasing order of HbA1c. Boxes show median values with upper and lower quartiles. Short horizontal lines and error bars in each box plot denote the mean values and standard deviations of individual cell measurements (n = approx. 40 per each group), respectively.

Our results show a strong correlation between HbA1c and dynamic fluctuations in RBC membranes, as shown in Fig. 4. The most noticeable alterations in diabetic RBCs can be found when the mean RBC membrane fluctuations of individuals are rearranged in the increasing order of the measured HbA1c levels. The mean membrane fluctuation of non-diabetic RBCs tends to decrease as the HbA1c level increases (Pearson correlation coefficient of −0.87 with a *p*-value of 0.02). In contrast, RBCs from diabetic patients exhibit significantly decreased membrane fluctuations, and the amplitude of dynamic membrane fluctuations are independent of the level of HbA1c. The mean values of membrane fluctuations for RBCs are 59.7 ± 6.2, 58.3 ± 4.9, 55.0 ± 5.4, 57.1 ± 7.4, 51.0 ± 6.8 and 49.6 ± 4.6 nm for healthy volunteers, and 45.1 ± 4.0, 46.9 ± 3.4, 48.8 ± 4.0, 47.6 ± 4.2, 43.0 ± 4.1 and 48.0 ± 5.0 nm for diabetic patients, in order of increasing HbA1c. This observed negative correlation between RBC membrane fluctuation and HbA1 is also conceptually in accordance with the report of a previous study, which reported a negative correlation between Hb1Ac levels and cell deformability indexes measured using a microchannel capillary model system^11^.

### Correlative analysis of individual RBCs

**Figure 5:**
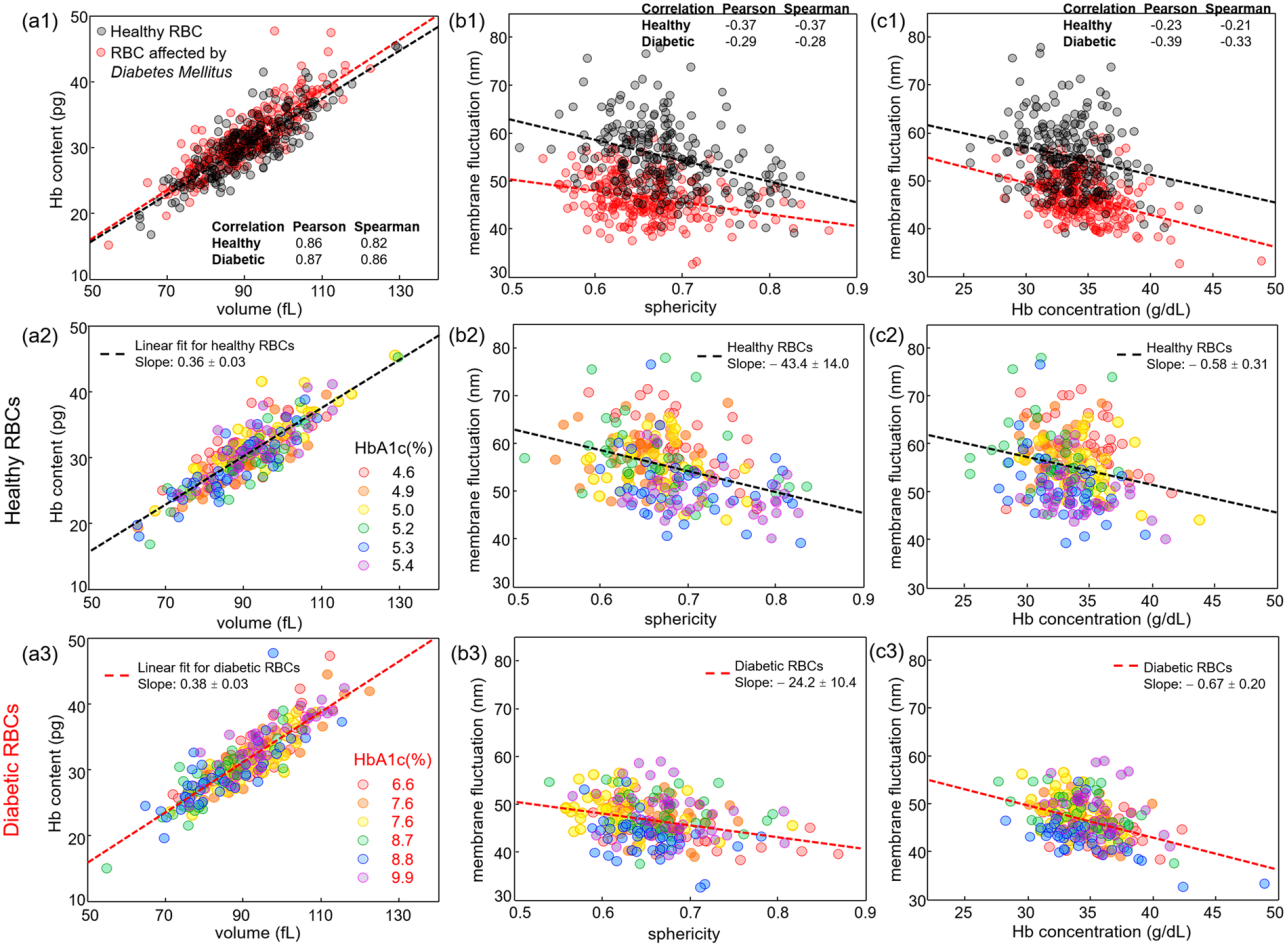
Scatter plots for various RBC parameters of non-diabetic and diabetic RBCs: (**a1**–**a3**) cell volume vs. Hb content, (**b1–b3**) sphericity vs. membrane fluctuation and (**c1**–**c3**) Hb concentration vs. membrane fluctuation. Each coloured circle denotes an individual RBC measurement. Scatter plots in the second (**a2**, **b2** and **c2**) and third rows (**a3**, **b3** and **c3**) represent RBC distributions of healthy and diabetic blood donors, respectively. RBCs from different individuals are presented in various colours according to HbA1c level. The dashed line in each figure represents a linear fit curve and the given slope covers 95% confidence interval. Pearson and Spearman coefficients for healthy and diabetic RBC groups are presented.

To fully exploit cellular alterations associated with diabetic complications, we performed correlative analyses among the retrieved parameters of individually measured non-diabetic and diabetic RBCs. There are four independent RBC parameters, since Hb content is obtained from Hb concentration and volume, and cell sphericity is directly linked to cell volume and surface area. Therefore, there exists a total of six pairs of independent parameters. Then, of total six pairs, scatter plots of RBC volume vs. Hb content, sphericity vs. membrane fluctuation, and Hb concentration vs. membrane fluctuation are presented and analysed in Fig. 5, respectively. This is because statistical analyses on the remaining pairs do not guarantee the significant correlations.

Statistical analysis using the linear regression model based on analysis of variance (ANOVA) shows that both healthy and diabetic RBCs share positive linear correlation between the cell volume and cytoplasmic Hb content, as shown in Fig. 5(a1–a3). The slopes of the linearly fitted curves for non-diabetic and diabetic RBC populations are 36.4 ± 2.8 (with 95% confidence intervals) and 38.0 ± 2.8 g/dL, implying the respective intracellular Hb concentrations. Figure 5(a2) and (a3) present Hb content vs. volume correlation maps of non-diabetic and diabetic RBCs, respectively, and the circles denoting individual cell measurement are designated with different colours according to the HbA1c level of the blood donors. Meanwhile, the statistical test based on the analysis of covariance (ANOCOVA) does not guarantee the significant difference in the slopes of the linear fit curves for healthy and diabetic RBCs (*p*-value = 0.42). Also, one notable point in Fig. 5(a1–a3) is that the slopes of the linear fit curves are both slightly higher than the corresponding mean Hb concentrations of both healthy and diabetic RBCs, we obtained earlier [Fig. 2(d)]. This is because the slope in a linear model essentially indicates a correlation between two variables, whereas the simple average largely depends on the absolute values of the variables itself. In other words, the mean Hb concentration is affected not only by the slope of the linear fit curve but also by the *y*-axis intercept, so that the negative intercept values for healthy and diabetic RBCs make the mean Hb concentrations less than the slope counterparts.

Sphericity and membrane fluctuation of non-diabetic and diabetic RBCs have a negative correlation with both *p*-values less than 0.001, as shown in Fig. 5(b1). Increased sphericity with reduced membrane fluctuations of RBCs associated with intracellular ATP depletion might account for the major portion of this overall negative correlation^39^. In Fig. 5(b2) and (b3), the membrane fluctuation vs. sphericity correlation maps of non-diabetic and diabetic RBCs are respectively presented with the HbA1c-dependent coloured circle, so that cell distribution changes in relation to HbA1c level can be effectively addressed. In particular, it seems that the cell distribution of non-diabetic RBCs in Fig. 5(b2) approaches to the lower-right end of the correlation map as HbA1c increases. The correlation maps show that RBCs from patients with diabetes exhibit significantly decreased mean membrane fluctuations compared to the healthy subjects in all ranges of sphericity. The scattered distribution of diabetic RBCs in Fig. 5(b3) with the significantly smaller slope of the linear fit curve than that of normal RBCs (*p*-value = 0.03) implies that the response of deformability to changes in cell sphericity is more insensitive.

Also, the correlation map of Hb concentration vs. membrane fluctuation in Fig. 5(c1) indicates that both non-diabetic and diabetic RBCs have a trend of exhibiting lower membrane fluctuations with higher Hb concentrations (*p*-values of less than 0.001 for both healthy and diabetic RBCs). Decreased deformability due to the elevated cytoplasmic viscosity of RBCs^45^ may account for this overall negative correlation between Hb concentration and membrane fluctuation, regardless of the RBC type. However, the statistical analysis does not reject the null hypothesis of same slopes of the linear fit curves for distributions of healthy and diabetic RBCs.

## Discussion

### Morphological and biochemical alterations of diabetic RBCs

To our knowledge, our study is the first to present the simultaneous measurements of 3-D morphological, biochemical, and mechanical properties of diabetic RBCs at the individual cell level. Previously, a few attempts have been made to investigate diabetic RBCs using quantitative phase imaging techniques. However, none of the previous work has comprehensively measured these parameters; all the previous works have been limited to 2-D phase imaging, where biochemical and morphological information are coupled to each other, and cannot be retrieved^54, 55^.

In this work, our results show that diabetic RBCs do not have statistically different morphological and biochemical parameters when compared to those of non-diabetic RBCs. Interestingly, one notable point arises when applying the parametric statistical analysis, called Student’s *t*-test to the integrated RBC data of all subjects (240 healthy and diabetic RBCs). In this case, diabetic RBCs have statistically higher cytoplasmic Hb concentration (*p*-value < 0.001) and content (*p*-value < 0.05), surface area (*p*-value < 0.01), and smaller sphericity (*p*-value < 0.001) than healthy RBCs. Observed discrepancies between these two statistical methods are in fact mutually compatible and lead to the following conclusion; although mean Hb concentration and content, and surface area of RBCs from six diabetic subjects are statistically higher than those of six healthy subjects, it is inconclusive whether the direct cause is diabetes. The non-enzymatic glycosylation, i.e. glycation, of Hb molecules^51, 52^ and membrane proteins^6^ by chronically elevated blood glucose content might be actually associated with those slight alterations in morphological and biochemical parameters of diabetic RBCs. Of course, for verification, it is essential to increase the number of subjects participating the experiment. Meanwhile, correlative analyses performed between those RBC parameters and the HbA1c level do not support clear dependency of morphological and biochemical parameters on HbA1c (Fig. 5 and Supplementary Figure S1).

### Diminished membrane fluctuation of diabetic RBCs: reduced deformability

Even between RBCs of similar values of sphericity or Hb concentration, considerable differences in membrane fluctuation of non-diabetic and diabetic RBCs were observed, as shown in Fig. 5(b1) and (c1). This implies that the mechanical alteration of the RBC membrane cortex is a leading cause of the diminished membrane fluctuations of diabetic RBCs. Widely known negative correlations between cell sphericity, Hb concentration and membrane fluctuation are also confirmed both in non-diabetic and diabetic RBCs. Mechanical alterations of diabetic RBC membranes are thought to be the consequences of the irreversible glycation process of the cell membrane and intracellular proteins rather than the instant effects of high blood glucose. This is because previous research with the *in vitro* treatment of glucose on diluted blood does not significantly change the RBC deformability^13^. The observed negative correlation between the mean membrane fluctuation of RBCs and HbA1c level in Fig. 4 also supports this view. Presumably, either an increase in membrane stiffness through glycation of band proteins comprising the spectrin network^10^ and membrane proteins^6^, or an accruing of peroxidation damages to the lipid membrane^7, 56^, or maybe both, account for the major portion of the reduced deformability of diabetic RBCs.

## Conclusion

We have quantitatively and non-invasively characterised the morphological, biochemical, and mechanical alterations of individual RBCs by diabetes mellitus. The HbA1c levels of subjects, which reflect one’s mean blood glucose concentration of the previous three months, were also determined using HbA1c analysers. Accordingly, correlative analyses among the retrieved individual RBC parameters and HbA1c level were performed to search for unique features which cannot be addressed by conventional techniques incapable of simultaneous measurement of those parameters.

Our optical measurements on individual RBCs from a total of 12 healthy and diabetic subjects show that normal and diabetic RBCs do not exhibit statistically different morphological and biochemical RBC parameters. On contrary, dramatic alterations in diabetic RBCs are found in their membrane fluctuations; diabetic RBCs exhibit membrane fluctuations significantly lower than those of non-diabetic RBCs. Successively performed correlative analyses further show that healthy donors tend to have RBCs of higher sphericity and lower membrane fluctuation, with a higher HbA1c value. These observations imply that hyperglycaemia associated with diabetes severely impairs RBC deformability by remodeling mechanical properties of the cell membrane.

With capabilities enabling quantitative measurements of individual microscopic cells under biocompatible conditions, we envision that QPI methodologies could shed light on unresolved areas of diabetes mellitus. A miniaturised QPI unit^57–59^ can convert conventional bright field microscopy into a QPI system by simply attaching the unit to a microscope body with light source adjustment. QPI techniques employing DMD-based illumination schemes^60^ or graphic-processing units^61^ allow real-time visualisation of a 3-D microscopic object, and are expected to become an attractive imaging tool for investigating live cell dynamics. A white-light diffraction tomography^62^ is free from the problem of speckle noise by using a low-coherent light source and can obtain 3-D images of individual cells with high axial resolutions. Furthermore, recently implemented QPI-based real-time blood testing method^63^ or tomographic flow cytometry^64^ is expected to play an important role in future clinical research fields because these techniques enable high-throughput analysis of individual cells.

## Materials and Methods

### Blood sample preparation and ethics statement

3 mL of blood was collected from six patients with type 2 diabetes mellitus by venipuncture and transferred into EDTA anticoagulant tubes at Asan Medical Centre, Seoul, Republic of Korea. CBC tests were instantly performed using an XE-2100 automated haematology analyser (Sysmex Co., Kobe, Japan), from which mean corpuscular Hb, mean corpuscular Hb concentration and mean corpuscular volume with RBC distribution width of individual diabetic patients were obtained. Independently, HbA1c was determined based on high-performance liquid chromatography using a TOSOH G8 analyser (Tosoh Co., Tokyo, Japan). For optical measurement, blood samples were delivered to a certified laboratory (IRB project: KH2015–37) at KAIST (Korea Advanced Institute of Science and Technology), Daejeon, Republic of Korea, within four hours of blood collection. All blood gathering protocols performed at Asan medical centre were approved by the ethics committee of the University of Ulsan, Ulsan, Republic of Korea (IRB project: #IRB-13-90). For the purpose of comparison, control blood was drawn by venipuncture from six healthy blood donors with the same blood collection protocols at a health clinic (the Pappalardo Centre at KAIST. An XT-2000i (Sysmex Co., Kobe, Japan) haematology analyser and a Cobra Integra 800 chemistry analyser (Roche Diagnostics, Risch-Rotkreuz, Switzerland) were used for the CBC test and determination of HbA1c, respectively. All the experimental protocols for the control blood donors were approved by the institutional review board of KAIST (IRB project: KH2015-37). Blood samples were stored in a refrigerator of 4 °C and optically measured using cDOT within 24 hours of blood gathering. In relation to the storage process, a recent study reported a result of invariances in mean membrane fluctuation of stored RBCs at 4 °C within 2 or 3 days after blood collection^36^. All the blood samples were collected as part of a regular course of patient care in Asan Medical Centre, and we selected patients who had given informed consent for using their archival tissues for genetic testing. All data was de-identified. All methods were performed in accordance with the relevant guidelines and regulations.

### Optical measurement on individual RBCs

To optically measure the individual RBCs, 3 μL of collected blood was diluted by a factor of 300 with Dulbecco’s phosphate buffered saline (Gibco®, New York, U.S.A.). The blood dilution process not only makes individual RBCs be unaffected by the surrounding cells during the measurement, but also homogenises the extracellular condition like osmolality or ion concentration by adjusting the medium characteristics to those of the buffer solution. Diluted blood suspensions were then sandwiched between two coverslips (24 × 50 mm, C024501, Matsunami, Ltd, Japan) and loaded on a sample stage of the inverted microscope body. After 20 min of waiting for cell settlements on a bottom coverslip, 40 RBCs for each healthy donor or diabetic patient were measured. In measuring RBCs, only gently sedimented RBCs of discocytes were selected. After excluding erroneously measured RBCs, 40, 40, 40, 38, 39 and 40 control RBCs of six healthy donors, and 40, 40, 40, 40, 40 and 40 diabetic RBCs of six diabetic patients were analysed respectively for the study of diabetic effects on characteristics of individual human RBCs.

### Statistical analysis

Specified *p*-values in the manuscript were calculated based on a non-parametric statistical analysis, so-called a Wilcoxon rank-sum test comparing RBC parameters between control and diabetes RBC groups, unless otherwise specified in the manuscript. In applying the test, red cell parameters of 40 RBCs assigned to each subject were averaged and regarded as one data point. In correlative analyses, ANOVA based linear regression model was employed to obtain linear fit curves for both control and diabetic RBCs. All the numbers following a ± sign in the linear regression model cover a 95% confidence interval. In the process of statistically comparing the correlations between different cell parameters of healthy and diabetic RBCs, one-way ANOCOVA was used. To further investigate the correlative nature of various RBC parameters along the HbA1c level, Pearson (linear correlation) and Spearman (monotonous correlation) correlation coefficients were calculated. Throughout the manuscript, all retrieved RBC parameters were given as mean ± SD and a *p*-value of less than 0.05 was regarded as statistically significant.

### Common-path Diffraction Optical Tomography (cDOT)

For simultaneous measurements of both 3-D RI tomograms and 2-D membrane fluctuations of RBCs, we employed cDOT^26^. A diode-pumped solid-state laser (λ = 532 nm, 50 mW, Cobolt Co., Solna, Sweden) was used as a light source. The laser impinges on a sample loaded on the stage of an inverted microscope with various illumination angles, which is controlled using a dual-axis galvanometer mirror (GM1; GVS012/M, Thorlabs, U.S.A.). A condenser objective lens [UPLFLN 60x, numerical aperture (NA) = 0.9, Olympus Inc., San Diego, CA, U.S.A.] was used for illuminating a sample with a broad range of impinging angles, and the high-NA objective lens (PLAPON 60x, oil immersion, N.A. = 1.42, Olympus Inc., San Diego, CA, U.S.A.) was used to collect diffracted light from a sample. The other galvanometer mirror (GM2), located at the conjugated plane of the sample, was synchronised with GM1 so that the laser beam after GM2 always followed the same path regardless of the laser illumination angle.

The rest of cDOT constitutes common-path laser interferometry. Using a diffraction grating (92 grooves/mm, #46-072, Edmund Optics Inc., NJ, U.S.A.), the laser beam after GM2 was divided into various diffracted orders. The brightest 0^th^ order diffracted beam was then spatially filtered by passing through a small pinhole (∅25 μm, P25S, Thorlabs, U.S.A.) located at the Fourier plane, and served as a reference arm in off-axis interferometry. The 1^st^ order diffracted beam containing the sample information served as a sample arm. Then, the spatially modulated interferograms of the sample were formed at the image plane, which was recorded by the high-speed sCMOS camera (Neo sCMOS, ANDOR Inc., Northern Ireland, U.K.). The total magnification of the system was 250x, contributed from both the 60x imaging objective lens and the additional telescopic 4-*f* imaging system. The field of view of cDOT was 14.3 × 13.31 μm^2^, which corresponded to 528 × 512 camera pixels with a pixel size of 6.5 μm

In our experiments, to obtain a single tomogram of individual RBCs, a set of interferograms obtained at 300 different illumination angles were successively recorded at a framerate of 100 Hz. From the captured interferograms, the complex optical fields, consisting of amplitude and phase information, can be retrieved by using proper field-retrieval algorithms^31, 65^. Then, from a set of the retrieved complex optical fields with different illumination angles, a 3-D RI tomogram of the sample *n*(*x*, *y*, *z*) was reconstructed employing optical diffraction tomography^28^. To fill the missing cone information caused by the limited NAs of the lenses, the regularisation algorithm based on non-negativity criteria was employed^66^. There is also an alternative way to obtain 3-D cell tomogram that the sample is rotated under fixed light illuminations^67^, instead of illuminating the sample with lights at different incident angles. Detailed information about optical diffraction tomography including the Matlab code can be found elsewhere^29^. For the visualisation of the measured 3-D RI tomograms, commercial software (Tomostudio, Tomocube, Inc., Daejeon, Republic of Korea) was used.

To measure 2-D height profiles and membrane fluctuations of RBCs, the laser illumination was set to be perpendicular to the sample. Consecutive interferograms were then continuously recorded with the high-speed camera at a frame rate of 125 Hz for 2.4 sec, from which 2-D phase delay maps Δ*ϕ* (*x*, *y*, *t*) were retrieved using the field retrieval algorithm. 2-D RBC height profiles *h*(*x*, *y*, *t*) were then calculated according to the following relation:

1 $$h(x,y,t)=[\lambda /(2\pi \cdot \langle {\rm{\Delta }}n\rangle )]\cdot {\rm{\Delta }}\varphi (x,y,t)$$

### Analysis procedures for retrieving RBC parameters

All the retrieved RBC parameters include morphological (volume *V*, surface area *S* and sphericity *SI*), biochemical (Hb concentration [Hb] and Hb content), and mechanical (membrane fluctuation σ_h_) properties of individual RBCs.

Of the morphological parameters, *V* and *S* of an individual RBC can be directly obtained from the reconstructed 3-D RI tomogram. Sphericity, *SI*, a measure of sphere resemblance, is then calculated as a normalised volume-to-surface area ratio as follows:

2 $$SI={(6V)}^{2/3}{{\rm{\pi }}}^{1/3}/S.$$

To obtain [Hb] from tomographic measurements of RBCs, it needs to be noted that the RI difference between the cytoplasm and the surrounding medium is linearly proportional to the concentrations of intracellular non-aqueous solutes with a proportionality coefficient, α, which is a so-called refraction increment^32^. This relation can be written as follows:

3 $$\langle {\rm{\Delta }}n\rangle ={\langle n(x,y,z)\rangle }_{spatial}-{n}_{m}=\alpha [{\rm{Hb}}],$$

where *n*(*x*, *y*, *z*) is a spatial RI distribution of a sample, and *n*_*m*_ is a medium RI. For mixtures of various chemicals, α is determined as a linear combination of α values assigned for each chemical with weight coefficients of respective concentrations. The practical issue is whether there are detectable differences in values of α between RBCs from healthy controls and ones from diabetic patients. Related to this issue, it has been recently reported that the dramatic alterations in protein compositions and growing parasites at the late stage of malarial infection result in a significant discordance between the cytoplasmic oxy-Hb content and the phase-originated cellular drymass^68^. In the case of diabetes, however, the most prominent alterations in cell components by hyperglycaemia is the glycation of Hb molecules, an additional formation of stable ketoamine linkages on Hb, and it does not significantly alter the protein structures, except for adding glucose molecules at the terminal. In particular, the percentage of HbA1c to total Hb content can be elevated at most by 6 ~ 7% in diabetic patients, as compared with normal HbA1c range^5^. Therefore, given the fact that Hb constitutes more than 90% of normal RBC proteins, expected changes in value of α by diabetes are thought to be small enough not to be considered. Hence, α was set to have 0.18 mL/g^69, 70^ for RBCs from both healthy controls and ones from diabetic patients. Of course, the unexplained or unexpected effects of diabetic mechanisms on RBCs might be a potential source of error. Then, cytoplasmic Hb contents can be obtained by multiplying *V* by [Hb].

In this paper, the 2-D membrane fluctuation map, σ_h_ (*x*, *y*), is defined as a temporal standard deviation of mean height profiles of RBCs, *h*(*x*, *y*, *t*), and can be written as:

4 $${{\rm{\sigma }}}_{{\rm{h}}}(x,y)={[{\langle {(h(x,y,t)-{\langle h(x,y,t)\rangle }_{{\rm{temporal}}})}^{2}\rangle }_{{\rm{temporal}}}]}^{1/2}$$

The membrane fluctuation, σ_h_, is then a spatial average of the 2-D fluctuation map,

5 $${{\rm{\sigma }}}_{{\rm{h}}}={\langle {{\rm{\sigma }}}_{{\rm{h}}}(x,y)\rangle }_{{\rm{spatial}}}$$

over projection cell area, and serves as the intuitive measure of RBC deformability.

## Electronic supplementary material

Supplementary Information
