# Supplementary material for: Refractive index tomograms and dynamic membrane fluctuations of red blood cells from patients with diabetes mellitus
Source: Sci Rep. 2017 Apr 21;7:1039. doi: 10.1038/s41598-017-01036-4 (PMC5430658; doi:10.1038/s41598-017-01036-4)
Supplement: Supplementary file 1 — Supplementary Information [file 41598_2017_1036_MOESM1_ESM.pdf]

Refractive index tomograms and dynamic membrane fluctuations of red blood cells from patients with diabetes mellitus

SangYun Lee[1], HyunJoo Park[1], Kyoohyun Kim[1], YongHak Sohn[2,*], Seongsoo Jang[3,*], and YongKeun Park[1,4*]

[1]Korea Advanced Institute of Science and Technology, Department of Physics, Daejeon 34141, Republic of Korea
[2]Department of Laboratory Medicine, Eulji University Hospital, Daejeon 35233, Republic of Korea.
[3]Asan Medical Center, University of Ulsan, College of Medicine, Department of Laboratory Medicine, Seoul 05505, Republic of Korea
[4]Tomocube Inc., Daejeon 34051, Republic of Korea

[*]Corresponding Authors: medsohn@eulji.ac.kr, ssjang@amc.seoul.kr, and yk.park@kaist.ac.kr
Keywords: red blood cell, erythrocyte, deformability, diabetes mellitus, quantitative phase imaging, optical diffraction tomography.

Supplementary Information

Correlations between retrieved RBC parameters and HbA1c levels of individuals

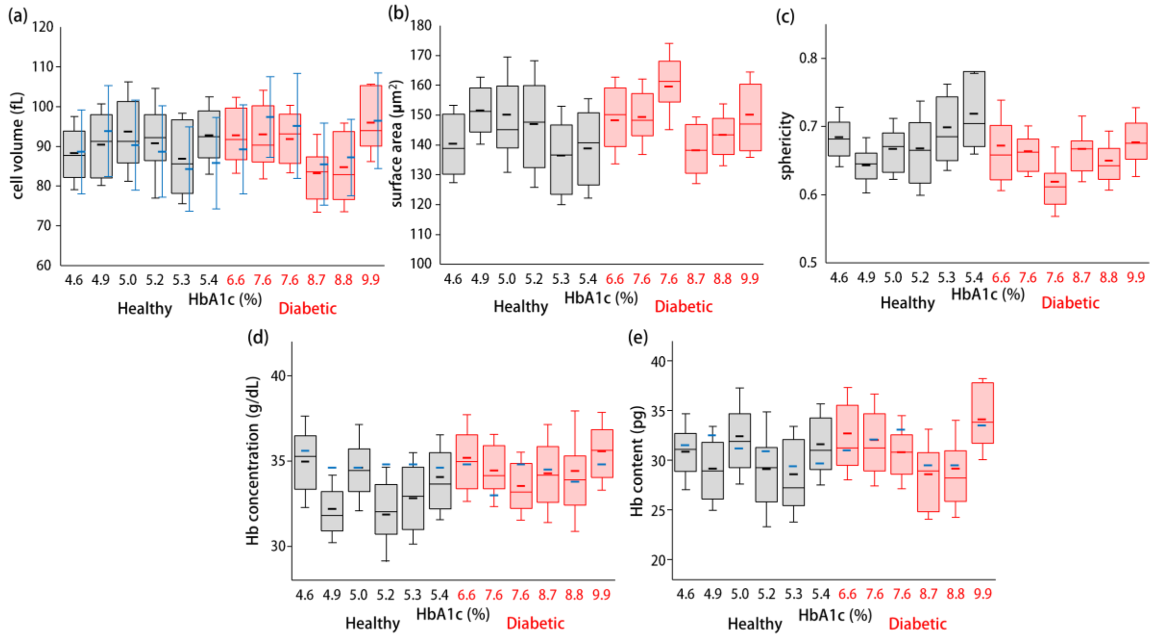

Fig. S1. The box plots for morphological and biochemical parameters of measured RBCs from individual healthy controls and diabetic patients in increasing order of HbA1c levels: (a) volume, (b) surface area, (c) sphericity, (d) Hb concentration, and (e) Hb content. Boxes, median values with upper and lower quartiles. Error bars in each box plot denote standard deviations and short horizontal lines represent mean values. Corresponding hemorheological parameters obtained from CBC results are presented in blue lines left to each box plot: MCV with RDW for cell volume, MCHC for Hb concentration, and MCH for Hb content, respectively.

For detailed investigations to clarify the effects of hyperglycemia on RBC characteristics, we conducted correlative analyses between the retrieved red cell parameters and the measured HbA1c levels of individuals, as shown in Fig. S1. In interpreting box plots, whisker and short thick horizontal line respectively denote sample SD and mean value of the retrieved RBC parameters. Besides, black and red box plots are for healthy blood donors and for patients with diabetes, respectively.

The box plots in Fig. S1(a) do not show clear dependence of mean RBC volume of individuals on HbA1c level. The mean RBC volumes are 88.3 ± 9.2, 90.4 ± 10.3, 93.7 ± 12.4, 90.8 ± 13.8, 86.9 ± 11.4, and 92.8 ± 9.7 fL for the healthy, and 92.8 ± 9.6, 93.0 ± 11.1, 91.8 ± 8.5, 83.2 ± 9.8, 84.7 ± 11.1, and 95.9 ± 9.7 fL for diabetic patients, with increasing levels of HbA1c. The blue horizontal line with an error bar right to each box plot denotes MCV with RDW-SD from the CBC test. These two independent measurements of mean RBC volume indeed coincide with high precision. In cases of surface area and sphericity, it also seems hard to find clear relations in between from the box plots in Figs. S1(b) and (c). The mean surface area and sphericity of measured RBCs for individuals with increasing levels of HbA1c are 140.4 ± 12.9, 151.5 ± 11.3, 150.2 ± 19.3, 147.1 ± 21.2, 136.5 ± 16.5, 138.8 ± 16.6 (the healthy), 148.2 ± 14.5, 149.5 ± 12.6, 159.7 ± 14.4, 138.2 ± 11.1, 143.4 ± 10.3, and 150.1 ± 14.3 μm^2 (diabetic patients), and 0.68 ± 0.06, 0.64 ± 0.04, 0.67 ± 0.04, 0.67 ± 0.07, 0.70 ± 0.06, 0.72 ± 0.06 (the healthy), 0.67 ± 0.07, 0.66 ± 0.04, 0.62 ± 0.05, 0.67 ± 0.05, 0.65 ± 0.04, and 0.68 ± 0.05 (diabetic patients), respectively.

When it comes to the cytoplasmic RBC parameters, intracellular Hb concentration and content of diabetic patients are slightly higher than those of healthy volunteers in partly HbA1c-independent manner. In this connection, variances in non-glycemic factors among individual subjects like

erythropoiesis or RBC turnover rate seem to precede the minute effects of Hb glycation. The mean Hb concentration and content for RBCs of healthy and diabetic blood donors are 35.0 ± 2.7, 32.2 ± 2.0, 34.6 ± 2.5, 31.9 ± 2.8, 32.8 ± 2.7, 34.1 ± 2.5 (the healthy), 35.2 ± 2.5, 34.4 ± 2.1, 33.5 ± 2.0, 34.3 ± 2.9, 34.4 ± 3.5, and 35.6 ± 2.3 g/dL (diabetic patients), and 30.9 ± 3.8, 29.2 ± 4.2, 32.4 ± 4.9, 29.1 ± 5.8, 28.6 ± 4.8, 31.6 ± 4.1 (the healthy), 32.7 ± 4.6, 32.0 ± 4.6, 30.8 ± 3.7, 28.6 ± 4.5, 29.1 ± 4.9, and 34.1 ± 4.1 pg in increasing order of the HbA1c level, respectively. The MCHC and MCH of individuals from CBC tests are also presented with blue thick lines in Figs. S1(d) and (e).
